# Patients’ interpretations of individual PROMIS-10 Global Health items: a cognitive interview study in high-impact chronic pain

**DOI:** 10.1007/s11136-026-04161-8

**Published:** 2026-02-01

**Authors:** Emily Sophia Madley, Henrik Bjarke Vaegter, Line Marie Saugmann Razinak, Daniel Broholm, Sophie Lykkegaard Ravn

**Affiliations:** 1 Pain Research Group, Pain Center, University Hospital Odense, Heden 7-9, Indgang 200, 5000 Odense C, Denmark; 2 Department of Clinical Research, Faculty of Health Sciences, University of Southern Denmark, Odense, Denmark; 3 Pain Center Middelfart, University Hospital of Southern Denmark, Middelfart, Denmark; 4 Department of Psychology, Faculty of Health Sciences, University of Southern Denmark, Odense, Denmark; 5 Specialized Hospital for Polio and Accident Victims, Roedovre, Denmark

**Keywords:** Chronic pain, PROMIS-10 Global Health, Content validity, Qualitative interviews, Quality of life

## Abstract

**Purpose:**

The purpose of this study was to explore the content validity of the Danish version of the PROMIS-10 Global Health questionnaire in patients with high-impact chronic pain by (1) identifying problematic items and reasons for misunderstandings between participants’ interpretations and the intended meaning, and (2) exploring how participants understand and interpret the items.

**Methods:**

Individual cognitive interviews were conducted with participants with high-impact chronic pain referred to two Danish Pain Centers using a structured step-by-step interview approach. The first objective was analyzed mainly using a deductive structured approach, while the second objective was analyzed mainly using an inductive thematic approach focusing on participants’ perspectives.

**Results:**

Participants (n = 19) generally understood the items as intended but showed 21% and 11% non-congruency for items 8r (fatigue) and 7r (pain intensity), as they responded based on a longer timeframe than “the past 7 days”. Overall, participants interpreted the items through a physical, psychological and social perspective. Further, items 5 (social activities and relationships) and 6 (daily physical activities) were perceived to overlap thematically with item 9r (social activities and roles). Additionally, the provided examples in items 5 and 6 influenced responses, and some deemed item 7r irrelevant, as it did not reflect their pain experiences.

**Conclusion:**

The findings contribute to the validation of PROMIS-10 Global Health in patients with high-impact chronic pain by providing insights into how participants understand and interpret the items, highlighting areas for minor refinement. Further qualitative research on the validity of PROMIS-10 Global Health in this population is needed.

## Abstract (plain language summary)

People living with high-impact chronic pain are often asked to complete questionnaires to assess their health and quality of life. One widely used tool is the PROMIS-10 Global Health questionnaire. However, it is not clear whether the questions fully reflect the experiences of patients with high-impact chronic pain. In this study, 19 patients with high-impact chronic pain were interviewed to explore how they understood and interpreted the questionnaire. Most questions were understood as intended, but several challenges emerged. In particular, questions about fatigue and pain intensity were often answered based on a longer time period than the stated “past 7 days”. Some questions were experienced as overlapping, for example, those about social roles and relationships, while examples provided in certain items strongly influenced how participants responded. Furthermore, some participants found that the pain question did not capture the complexity of their pain experience. These findings suggest that, while the PROMIS-10 questionnaire is generally appropriate for patients with high-impact chronic pain, certain items could be improved to better match patients’ lived experiences. The study highlights the importance of including patient perspectives when validating health questionnaires to ensure they are relevant, clear, and meaningful.

## Introduction

High-impact chronic pain (HICP) is defined as persistent pain and substantial related limitations in daily life activities for more than 3 months and affects 5–15% of the population [1]. Patients with HICP are more likely to experience mental health challenges, difficulties with personal care, cognitive impairments, social participation, and fatigue [2] as well as increased reliance on healthcare services compared to both the general population and those with less severe chronic pain [3, 4]. Also, HICP results in poorer health-related quality of life (HRQOL) [2, 5, 6]. HRQOL reflects the relationship between health and quality of life and is defined as an individual’s well-being and self-perceived functioning across social, mental, and physical domains of health [7]. HRQOL is one of the most important treatment outcomes for patients with HICP [6] and among the core outcome domains essential to measure in studies of chronic pain treatment [8].

HRQOL is often assessed as a Patient-Reported Outcome Measure (PROM). A PROM is a patient-administered questionnaire designed to capture the concepts and outcomes relevant to the target population [9]. One widely used PROM to measure HRQOL is the Patient-Reported Outcomes Measurement Information System Global Health Survey (PROMIS-10 GH). PROMIS-10 GH is a 10-item questionnaire developed to evaluate physical and mental health from the patient’s perspective and has been applied across multiple chronic health conditions, including chronic pain [7, 10].

Establishing content validity of a PROM is essential to ensure that the questionnaire adequately captures the relevant content and the full range of attributes that define the construct being measured [9, 11]. While the PROMIS-10 GH questionnaire has been quantitatively validated in various populations [12, 14], there is a lack of qualitative research examining its content validity, both in general and specifically in patients with HICP. Validation of PROMs is a multi-step process [9], where cognitive interviewing contributes to the assessment of content validity by exploring how respondents understand, interpret, and respond to the individual items. Hence, cognitive interviews can identify and address potential problems in item interpretation, relevance, and comprehensibility and can explore patients’ subjective understandings of the items [15, 16]. Cognitive interviewing is therefore an important and necessary step in the process of establishing the content validity of PROM, such as PROMIS-10 GH and in supporting its effective use in clinical practice and research [17]. In addition, cognitive interviews play a key role in the cross-cultural adaptation and validation of PROMs. When a questionnaire is translated into another language, cognitive interviews can identify potential issues related to language, cultural relevance, and conceptual understandings, ensuring that items are interpreted as intended across cultures and populations [18].

To address this critical knowledge gap, we conducted this study to provide essential insight into patient perspectives on the Danish version of PROMIS-10 GH by performing cognitive interviews with patients with HICP. Specifically, our two objectives were: (1) To identify potential problematic items and causes of misunderstanding by exploring the correspondence between the patients' interpretations and the intended meanings of the items in PROMIS-10 GH and (2) to explore the patients' subjective interpretations of the items in PROMIS-10 GH in relation to their life experiences.

## Methods

### Study design

This pre-registered study (10.17605/OSF.IO/5724P) was a qualitative interview study, where we conducted individual, face-to-face, semi-structured cognitive interviews with patients with HICP referred to two specialized university hospital pain centers in Denmark.

### Ethics and approvals

The study was registered with the Discretion Secretariat at Odense University Hospital on the 24th of October 2023 (journal no. 23/48829). According to Danish law, ethical approval was not required as the study was based on interviews and patient-reported data without conducting or testing an experiment or intervention [19]. The study followed the principles of the Declaration of Helsinki [20], where all provided written consent and were informed that participation was voluntary and would not affect treatment. The study complied with the General Data Protection Regulation; data were pseudonymized and securely stored [21].

### PROMIS-10 GH

The PROMIS-10 GH v1.2 questionnaire is designed to evaluate physical and mental health status through 10 items [10]. It encompasses physical, psychological, and social domains, and each item is rated on a 5-point Likert scale, except for item 7r, which uses a 0–10 scale [22]. Upon completion, T-scores for physical and mental health can be calculated [21] and standardized to the US general population (mean = 50, SD = 10), where higher scores indicate better HRQOL [20]. The physical health score is derived from items 3 (physical function), 6 (daily physical activities), 8r (fatigue), and 7r (pain intensity (reversed scored)). The mental health score is derived from items 2 (quality of life), 4 (mental health), 5 (social activities and relationships), and 10r (emotional problems). Hence, items 1 (general health) and 9r (social activities and roles) are not included in the calculation of the T-scores [23]. In this study, the Danish version of PROMIS-10 GH was used and obtained from HealthMeasures [24, 25]. HealthMeasures state that the translations are developed through a standardized linguistic validation process, including forward- and backward-translation, multiple expert reviews, harmonization across languages, and cognitive debriefing with native speakers of the target language [25].

### Recruitment and participants

Participants for the study were recruited from the waiting lists of two Danish university interdisciplinary pain centers, the pain center at Odense University Hospital [26] and the pain center at University Hospital of Southern Denmark [27]. Criteria for referral to the pain centers were HICP and ≥ 18 years of age. Participants were required to be proficient in Danish.

As part of the assessment and treatment at the two pain centers, patients complete a clinical questionnaire via the electronic registry PainData [5]. An additional item was included to obtain consent for contact. Patients who consented were contacted by phone by ESM, who provided further study information. Interested patients were invited to participate, and interviews were scheduled at one of the pain centers. Participation was confirmed by email, and recruitment occurred on a rolling basis. Purposive sampling ensured a diverse sample [15] and sufficient information power [28], considering gender, age, ethnicity, and pain location. The target sample size was 15–20 participants, in line with recommendations [16].

### Data collection

Data was collected through cognitive interviews using the Three-Step Test Interview method (TSTI) [29] and by ESM, a female master's student in Public Health, under supervision from HBV and SLR, who had previous experience with the method. Prior to data collection, ESM also received training in the method by conducting three pilot interviews. A pilot-tested interview guide with structured open-ended questions based on TSTI was used. Participants could bring a relative, who was asked to remain silent to avoid influencing the interview. Each interview lasted approximately 60 min and was audio-recorded. Beforehand, participants were informed about the study’s purpose, the TSTI procedure, and that ESM was a master’s student exploring their understanding of PROMIS-10 GH, emphasizing that there were no right or wrong answers.

To familiarize participants with the TSTI method [29], two printed practice questions were provided at the start of the interview to help participants practice the think-aloud process in step one, which can feel unnatural [15]. The questions, selected from the Major Depression Questionnaire (ICD-10), addressed reduced appetite and lack of interest in daily activities [30]. They were chosen to avoid content overlap with the PROMIS-10 GH items.

After the two practice questions, the interview began, where the participants were provided with a printed version of the Danish version of PROMIS-10 GH. In one online interview, the participant had printed the questionnaire and relevant documents in advance. During the first TSTI step, primary data was collected through observation as participants filled out the questionnaire while verbalizing their thoughts [29]. ESM took notes on their behavior. In the second step, these notes were used to ask clarifying questions through follow-up probing [29]. In the third step, participants were asked to share experiences, explaining reasons for their responses, and providing personal examples [29]. This involved ESM probing reasons behind specific responses, such as asking why a participant had chosen to answer 'x' for a particular item and encouraging them to provide personal examples illustrating their responses. As an extension of step three in the TSTI method, participants were encouraged to reflect on the concepts in the questionnaire and how these applied to their lives, aiming to capture personal narratives for deeper insight. All participants completed all steps of the TSTI. Interviews were transcribed verbatim, and participants were offered the opportunity to review the transcripts; however, no participants provided feedback.

### Data analysis

#### The first objective

The study’s first objective was analyzed using two approaches. The part concerning the correspondence between participants’ interpretations and the intended meanings of the PROMIS-10 GH items was analyzed mainly deductively using Coding Reliability Thematic Analysis [31], based on systematic coding through predefined categories. A coding framework inspired by Bunzli et al. [32] was applied, consisting of four categories: 1) the participant’s response was consistent with the intention of the item (congruent response), 2) the participant’s response was not consistent with the intention of the item (incongruent response), 3) the participant misunderstood the content of the response scale (confusion), and 4) the participant’s response was both incongruent and congruent (ambiguous) [32]. To ensure consistency within the research team, a coding manual was developed, outlining characteristics of congruent responses for each item based on literature from PROMIS development [33, 34] and WHO definitions [35]. The manual was piloted by having researchers from the team independently code two interviews, followed by group discussions to resolve discrepancies.

The analysis primarily drew on data from the first and second steps of the TSTI, which provided more direct insight into participants’ immediate reflections [29]. When needed, the third step was used to inform coding. All interviews were independently double-coded: ESM coded all interviews, while DB (PhD student) and LMSR (research assistant) each coded half. Discrepancies were resolved through discussion, and, if needed, by consulting the wider research group. After analysis, codes were quantified for congruency, incongruency, ambiguity, and confusion, and items with > 50% non-congruent responses were considered problematic.

The other part of the first objective regarding causes for non-congruency was analyzed using a more inductive approach, where ESM systematically analyzed responses to elucidate the underlying causes linked to responses coded as non-congruent. This was done by gathering all non-congruent responses for each item, reviewing them, and making notes on the reasons for non-congruence. These reasons were then analyzed to identify specific underlying causes that led to responses being coded as non-congruent.

#### The second objective

The second objective, regarding participants’ interpretations and understandings of PROMIS-10 GH items in relation to their own life experiences, was analyzed by ESM using a mainly inductive approach with Reflexive Thematic Analysis (RTA) [36]. The analysis followed the six steps of RTA, emphasizing the iterative nature of theme development. As a novice in RTA, ESM approached the process with an awareness of its reflexive character, continuously engaging with the data to refine interpretations and remain open to unexpected patterns. In line with RTA principles, themes were not merely identified but actively constructed through iterative engagement with the data [36].

The analysis primarily drew on data from the third step of the TSTI, which focused on participants’ reflections and interpretations, providing more in-depth and nuanced insights [29]. All transcripts were read and re-read to ensure thorough familiarity with the dataset, then organized by each PROMIS-10 GH item to facilitate a systematic analysis. Once organized, the formal coding process began, conducted sequentially for each item. For each item, steps two to five of RTA were completed before proceeding to the next. Following these steps, transcripts were systematically reviewed and coded inductively, with codes serving as concise labels capturing both explicit participant statements (semantic) and underlying meanings (latent). Patterns across codes were then analyzed to identify themes representing shared interpretations. These preliminary themes were subsequently reviewed and refined against the original transcripts for accuracy and coherence. In the final steps, themes were clearly defined, named, and illustrated with representative quotes to enhance clarity and readability [36].

## Results

The study included a total of 19 participants. Of these, 15 interviews were conducted at the pain center at Odense University Hospital, three interviews were conducted at the pain center at the University Hospital of Southern Denmark, and one interview was conducted online. Three participants chose to bring a relative for support, who was present in the interview room. The 19 participants had a demographic distribution of 15 women, a mean age of 51, a mean pain score of 7.5 on a 0 to 10 scale in the last 7 days and a mean pain duration of 118.35 months. Participants reported a variety of pain locations and had average T-scores of 32 for physical health and 41.2 for mental health on PROMIS-10 GH. The descriptive characteristics are presented in Table 1.

**Table 1:** Descriptive characteristics of patients with high-impact chronic pain (n = 19)

Variables	Total (n = 19)
Sex female (n)	15
Age in years (mean (range))	51 (27–68)
Ethnicity (n)	
Danish	18
Other	1
Pain Intensity from 0 to 10 in the last 7 days (mean (range))	7.5 (5–10)
Pain duration in months (mean (range))	118.35 (12–462)
Pain location (n)	
Local (ankle, hip)	2
Regional (spine, leg, arm, head)	10
Widespread (> 1 regions)	7
PROMIS-10 Global Health T-scores (mean (range))	
Physical health	32 (19.9–39.8)
Mental health	41.2 (28.4–53.3)

### The first objective

The results regarding the first objective are summarized in Table 2, where each item is presented alongside the percentage of congruent, incongruent, confused, and ambiguous responses. All participants completed the interviews, and there were no missing responses.

**Table 2:** Results from coding cognitive interviews regarding assessment of congruency levels in patients with high-impact chronic pain (n = 19)

Items	Congruent (%)	Incongruent (%)	Confused (%)	Ambiguous (%)
Item 1 (general health)	100	0	0	0
Item 2 (quality of life)	100	0	0	0
Item 3 (physical health)	100	0	0	0
Item 4 (mental health)	100	0	0	0
Item 5 (social activities and relationships)	100	0	0	0
Item 9r (social activities and roles)	95	0	0	5
Item 6 (daily physical activities)	100	0	0	0
Item 10r (emotional problems)	100	0	0	0
Item 8r (fatigue)	79	5	0	16
Item 7r (pain intensity)	89	0	0	11

The table describes the items in the PROMIS-10 Global Health questionnaire and indicates the percentage of responses categorized as congruent, incongruent, confused, or ambiguous

The results showed that no items in PROMIS-10 GH were identified as problematic using the pre-defined cutoff of 50%, as all items had 79% or more congruent responses. Most items had 100% congruent responses, with only three items having less. The analysis showed respectively 5%, 21%, and 11% non-congruency for items 9r (social activities and roles), 8r (fatigue), and 7r (pain intensity).

The analysis identified four causes of non-congruency. Most non-congruent responses were related to the timeframe specified for items 8r and 7r. According to the coding manual, a response was labeled non-congruent if participants clearly referred to experiences outside the “last 7 days” timeframe. Some participants referred to experiences from earlier periods, often because they overlooked the timeframe, which was displayed only above item 10r and not visible for the subsequent items. For others, the timeframe was perceived as irrelevant, as they reported that their responses would have been similar even if it had covered a longer period, such as the past year. Furthermore, cognitive challenges were noted in recalling experiences from the previous week. One response regarding item 9r (social activities and roles) was coded “ambiguous”, as the participant answered the item positively based on the assumption that their partner handled the roles for them, rather than their own ability to handle their role.

### The second objective

The results of the second objective were analyzed to explore the participants' subjective understandings and interpretations of the items in PROMIS-10 GH. The analysis resulted in one to three themes per item where the participants understood and interpreted the items through a biopsychosocial perspective. The results are presented in Table 3, where the participants' understandings and interpretations are outlined through the description of the identified themes for each item, the number of participants for whom the theme was identified and supporting quotes.

**Table 3:** Results from analysis of cognitive interviews in patients with high-impact chronic pain (n = 19)

Themes	Description	Supporting quotes
Item 1 (general health)		
Physical health (n = 19)	Participants’ interpretations were largely shaped by their physical condition, with chronic pain central to their assessment. Some focused solely on pain, while others considered additional illnesses, and the limitations pain imposed on daily life	*"Well, poor health is when you have pain, and good health is when you don't"* (P5)
Mental health (n = 11)	Mental health was also central to participants’ interpretations, as chronic pain often drained them mentally. For some, maintaining psychological resilience was essential for managing pain and preventing it from taking over daily life	*"So, my mental health also needs to keep up, and if it can't keep up, the pain can't stay manageable either, it takes over and steers everyday life"* (P11)
Social participation (n = 7)	The ability to engage with family, friends, and work influenced some participants' interpretations, as social connections were essential to their general health	*"It’s some of those social things that we also really enjoy in my family, you know. You just can’t… I mean, if we’ve been on some kind of outing, for example, well then you can’t"* (P13)
Item 2 (quality of life)		
Meaning in life (n = 11)	Quality of life was interpreted as having purpose and meaningful social connections. Participants emphasized the importance of feeling valued and having personal fulfillment	*"For me, it's about asking, well, what makes life worthwhile? It's both that I matter to someone, and that someone matters to me. And quality of life is also about what I put into my life, what I choose to fill my life with. So, I think it's also about the values one has"* (P2)
Freedom in life (n = 13)	Participants found the ability to act without restrictions from physical or psychological factors was central to quality of life. Pain-related limitations were linked to a poorer quality of life	*"Well, quality of life, I think, is being able to say, today I feel like doing this or that, or participating in this or that, just being able to choose freely and do whatever you want. That must be quality of life."* (P15)
Item 3 (physical health)		
Functional health (n = 19)	The participants interpreted physical health in relation to their ability to manage everyday physical tasks and the pain associated with them. They considered not only their capacity to carry out activities but also the impact of pain, the need for assistive devices, and support from family and friends	*"It takes half a day to vacuum because you vacuum one room and take a break, vacuum another room and take a break, and yeah, it just hurts all the time"* (P16)
Bodily health (n = 14)	Participants interpreted physical health in relation to injuries and pain in specific body areas. They also considered whether their health issues were chronic or acute when assessing their overall physical health	*"It hurts under the soles of my feet, it hurts in my, shoots through my knees and stuff like that, I still have neck tension, you know, it doesn’t bother me as much because my elbow makes so much noise and my arms and my hands"* (P17)
Item 4 (mental health)		
Mental energy (n = 16)	Participants interpreted mental health with their cognitive and emotional energy throughout the day. They viewed excellent mental health as the ability to function, engage socially, and handle daily demands without feeling mentally or emotionally drained	*"I don’t visit X (shopping mall) because I can’t handle it. Have you ever noticed that in each store, they actually play their own music, and then out in the hallway, they play something else entirely, and at the same time, there are so many people? I find it incredibly difficult to function in that environment"* (P2)
Perspective on life (n = 8)	Participants linked their mental health to their ability to maintain a positive outlook, focus on opportunities rather than limitations. Their perspective on pain, limitations, and the future played a key role in shaping their overall mindset and quality of life	*“I’m good at seeing the positive (…) I’m good at noticing the small positive things (…) well, I think I’m good at finding alternatives to things and figuring my way around them so that I still get something out of it"* (P19)
Item 5 (social activities and relationships)		
Participation in activities (n = 19)	Participants interpreted the item as their ability to engage in work, hobbies, family events, and social interactions. Higher activity levels were linked to positive social functioning, while limitations often led to reduced participation or withdrawal	*“I just really don’t like social events, like the birthday yesterday, I end up kind of sitting over in the corner. I’m just in pain. I do get through the day, and we have our meal, but I’m also the first to leave, saying I just need to go home now and relax"* (P4)
Social relations (n = 18)	Participants assessed the item based on the size and quality of their social networks and their ability to maintain meaningful connections. They prioritized close, supportive relationships while limiting broader social interactions due to the energy demands of being social with chronic pain	*"There are some people I have a good relationship with, and those I don’t feel contribute anything positive, well, they’re just, yeah. So, it’s important to me to have a good relationship with the people, who are close to me"* (P18)
Item 9r (social activities and roles)		
Social activities and relationships (n = 18)	Participants interpreted this item based on their engagement in work, leisure, family gatherings, and social roles. High ratings reflected frequent participation without issues like pain, while low ratings were linked to avoidance or cancellations. Some found the broad examples challenging, leading to feelings of inadequacy, and many noted overlaps with item 5, making differentiation difficult	*"Then I feel a bit guilty when it says clubs or associations, because I'm not part of any clubs or associations" (P17)* *"Well, it (social activities) is kind of the same again (as item 5), it’s the same"* (P8)
Duties and roles (n = 18)	Participants interpreted this item through their responsibilities at home and work, including tasks like cleaning, cooking, and supporting family activities. Many wished to contribute more equally but felt restricted by pain and its impact on daily life	*"In relation to my spouse, yes, I would like it to be more equal, but I end up feeling somewhat dependent because there are certain things I wouldn’t be able to do if X (husband) wasn’t there"* (P2)
Item 6 (everyday physical activities)		
Duties in everyday life (n = 19)	Participants interpreted the item in terms of their daily responsibilities, assessing their ability to perform tasks such as cleaning, walking, climbing stairs, and carrying groceries. A complete inability was understood as requiring full assistance, while high ratings indicated the ability to perform all the listed activities independently	*"(If responding “not at all”) Then I would need help with everything, yeah, going for a walk, there wouldn’t be any walks, going up stairs, you have to be really bad at walking to not be able to get up the stairs, carrying a shopping bag, couldn’t do that at all, and moving a chair, I think you wouldn’t be able to move a regular dining chair either, it’s really not possible"* (P11)
Item 10r (emotional problems)		
Negative thoughts (n = 18)	Participants interpreted this item through pain-related thoughts, often leading to frustration and irritation. Few experienced depressive thoughts, but many were frustrated by activity limitations and pain’s impact on daily life. Some found it unrealistic to answer “never”, associating it with emotional detachment	*"(If answering “never”) Then you’re never emotionally present, I think, so I don’t think that’s possible"* (P18)
Mental capacity (n = 7)	Participants often felt mentally overwhelmed by pain, making social and daily activities challenging. Many planned their week around limited mental capacity, frequently canceling events. This lack of energy led to irritability, strained relationships, and guilt, with a strong desire for more mental energy to engage fully in life	*"Then I have used too many of my resources, and I become irritable for the rest of the day because the pain is right there, lingering and adding an extra burden, which takes up a large part of my energy"* (P16)
Concerns for the future (n = 10)	Participants expressed concerns about the progression of their pain and its long-term impact on daily life. Many worried about their ability to work and support their family, leading to feelings of guilt about potentially becoming a burden	*"How does it (the pain) look in the future, how long can it go on, and how should it progress moving forward?"* (P16)
Item 8r (fatigue)		
Lack of sleep (n = 11)	Participants associated fatigue with poor or interrupted sleep due to pain, leading to daytime exhaustion and a cycle of disrupted rest. Many reported that insufficient sleep affected daily activities like cleaning, cooking, and driving. For some, “no fatigue” meant sleeping soundly through the night, while severe fatigue was linked to persistent sleep disturbances	*“Well, fatigue, I associate it with too little sleep"* (P19)
Physical and mental exhaustion (n = 17)	Participants described fatigue as both physical and mental exhaustion, affecting activities like driving, walking, and reading. Physical exhaustion made movement difficult, while mental exhaustion impaired concentration, decision-making, and mood	*"Well, it’s that sudden fatigue, especially, that can hit both mentally and physically, so even just moving something, and also in my head (…), that’s a really annoying thing"* (P7)
Item 7r (pain intensity)		
Pain intensity (n = 18)	Participants described their pain intensity as fluctuating throughout the week, making it difficult to summarize with a single number. They often averaged their highest and lowest pain levels, with good days ranging from 2–5 and worse days from 7–10, significantly impacting daily activities. Some suggested modifying the item to capture both extremes for a more accurate reflection of their pain experience	*“Yeah, but it’s because it fluctuates so much, I find it hard to rate it, because I can be down around 2–3, and then I can be at 10, so it’s really difficult to put a solid number on it"* (P19)

The table presents the items from the PROMIS-10 Global Health questionnaire, the themes associated with each item, number of participants for whom the theme was identified (n), a description of these themes and relevant supporting quotes

## Discussion

### Summary of findings

The results for the first objective showed that participants largely understood PROMIS-10 GH as intended, with over 79% congruent responses. Most items had 100% congruency; only items 9r (social roles), 8r (fatigue), and 7r (pain) showed 5%, 21%, and 11% non-congruent responses, mainly due to issues with the time frame. These included overlooking the time specification, finding it irrelevant, or having difficulty recalling past experiences. For the second objective, participants interpreted PROMIS-10 GH items from a biopsychosocial perspective, linked to one to three themes reflecting different aspects of HRQOL.

### Discussion of findings

#### First objective

While no qualitative studies to our knowledge exist for direct comparison of PROMIS-10 GH, the findings from this study support existing quantitative research, showing that PROMIS-10 GH aligns well with other questionnaires measuring HRQOL, including both general and condition-specific tools [12, 14].

While no items were found to be broadly problematic, some participants overlooked the time frame in items 8r (fatigue) and 7r (pain intensity). This appeared to occur primarily because the time frame instruction was presented only above item 10r, rather than being repeated for items 8r and 7r. Even when the time frame was noticed, some participants found it irrelevant or had difficulty summarizing their experiences within such a short period, with several reporting that a seven-day average did not reflect their fluctuating pain or fatigue. This may have implications for the clinical interpretation and use of the PROMIS-10 GH. These findings are consistent with a report by the Danish Health Data Authority, which noted that patients with chronic pain may miss the “past 7 days” reference in PROMIS-10 GH and recommended emphasizing the time frame more clearly within each item [37]. Also, the report noted similar concerns for item 10r (emotional problems), where recent events, such as holidays or personal loss, could distort responses [37]. To solve this challenge, the report proposed extending the time frame [37]. However, doing so may introduce recall bias [38]. The report also suggested allowing patients to report their minimum and maximum pain levels over seven days, which participants considered more meaningful [37]. This perspective was shared by some participants in the present study, who suggested that such a revision would better capture the fluctuating nature of pain. Previous research supports this, showing that individuals with chronic pain often find it difficult to summarize their experiences over short recall periods due to day-to-day symptom variability [39]. Moreover, studies comparing real-time assessments with recall ratings over a week or longer have found discrepancies in reported average fatigue and pain intensity [40]. In addition, studies report that recall ratings for periods of a week or more may be influenced by cognitive heuristics, such as recalling peak or recent experiences [41], and tend to indicate higher symptom levels than real-time assessments [39].

To address the challenges related to the timeframe in the PROMIS-10 GH, further research is needed. Based on the findings and literature, potential revisions could include: 1) explicitly integrating the timeframe into each item, 2) rephrasing to reflect real-time assessment, 3) allowing reports of minimum and maximum pain, or 4) removing the timeframe altogether to better capture typical pain and fatigue experiences and align these items with the rest of the items in PROMIS-10 GH. The latter option is supported by participants in this study, who noted that their responses, especially to item 7r, would likely have been the same if the timeframe had covered the past year.

One response regarding item 9r (social activities and roles) was coded as “ambiguous,” as the participant answered positively based on their partner’s ability to manage the roles on their behalf. As this issue was identified in only one participant and has not otherwise been highlighted as problematic, we do not see sufficient reason to revise this item.

#### Second objective

For the second objective, findings largely aligned with the Danish Health Data Authority report [37], while also revealing some differences. Both showed that pain strongly influenced responses to item 1, with participants focusing either solely on pain or also including other conditions. This study added that mental health and social participation also shaped interpretations. For item 2 (quality of life), both studies emphasized the role of meaningful relationships [37], and this study further identified “freedom in life” as an additional theme. Regarding item 3 (physical health), both studies found that chronic pain and other conditions impacted perceptions, with participants linking physical health to both functionality and bodily well-being [37]. Item 9r (social activities and roles) posed similar challenges in both the report [37] and this study due to the guiding effect of the provided examples, which influenced how participants evaluated their abilities. The same challenge applied to item 6 (daily physical activities). These findings suggest that items 9r and 6 could benefit from clarifying that the assessment should include broader aspects beyond the examples. Differences emerged in item 4 (mental health), where only the report found participants struggled to distinguish between mood and cognitive function [37]—an issue not present in this study. Similarly, patients found item 5 (social activities and relationships) difficult to interpret in the report [37], whereas participants in this study did not. However, both the report [37] and this study emphasized low energy for social activities and the importance of relationships with friends and family.

This study found thematic overlap among items 5 (social activities and relationships), 9r (social activities and roles), and 6 (daily physical activities), particularly regarding social participation, relationships, and duties. This raises questions about the necessity of item 9r, which appears to address themes already covered by items 5 and 6. Since item 9r is not included in the physical or mental health scoring of PROMIS-10 GH [23], its inclusion in the measure could be reconsidered. Participants also interpreted fatigue in item 8r as both physical and mental exhaustion, with mental fatigue influencing cognitive and emotional states. As Hays et al. found that this item correlated more with physical health, it was included in the physical health score [23]. However, the present findings suggest reconsidering whether item 8r should solely reflect physical health.

### Strengths, limitations, and methodological reflections

#### Recruitment and participants

Purposive sampling ensured diverse interpretations by including participants varying in sex, age, and chronic pain characteristics [42], thereby enhancing the study’s information power [28]. To ensure linguistic validity, only patients proficient in Danish were included. In several domains, participants reflected the broader HICP population, which is predominantly women with an average age of 50 years and chronic pain duration of approximately 120 months [5]. However, participants in this study reported slightly poorer physical health and slightly better mental health compared to the study population [5]. The low representation of ethnic minority participants (one person) may limit the representativeness of the Danish sample and should be considered when interpreting the findings [40]. Finally, recruitment via PainData may have introduced selection bias [38], as the comprehensive and cognitively demanding questionnaire could discourage less resourceful patients from participating.

#### Data collection

Cognitive interviewing provides insights into the overall quality and cross-cultural validity of PROMs by identifying issues in item interpretation, relevance, and comprehensibility [15, 16, 18], and is therefore essential part of establishing content validity [17]. In this study, cognitive interviews provided in-depth insights into responses to the PROMIS-10 GH, following recommended guidelines [15, 16]. Using the structured TSTI method enhanced data collection by capturing immediate reactions and clarifying response behaviors [29]. However, TSTI also poses challenges, as it creates an artificial setting that differs from normal questionnaire completion. Thinking aloud during the first step may feel unnatural and lead to incomplete reflections [15], and the interviewer’s presence might have influenced responses. The method is also resource-intensive, requiring lengthy interviews and deep reflection, which can cause cognitive fatigue and less reflective answers toward the end [29]. Finally, the online format of one interview limited observation of non-verbal cues, and the presence of relatives in three interviews may have influenced responses.

#### Data analysis

Overall, we argue that methodological triangulation [43] and double-coding of the deductive coding parts enhanced the credibility and consistency of the results, supported by a collaboratively developed and piloted coding manual. Also, applying the RTA method enabled a flexible and nuanced analysis of subjective experiences while maintaining researcher reflexivity [36]. However, the lack of explicit definitions for all PROMIS-10 GH items by the PROMIS group required referencing external literature, which may have led to discrepancies between the coding framework and the PROMIS framework. Furthermore, conducting a deductive analysis first may have influenced the inductive approach, introducing potential researcher bias [44].

## Conclusion and future directions

To our knowledge, this is the first study to use cognitive interviews to contribute to the validation of PROMIS-10 GH. Overall, participants understood and interpreted the items as intended, with only minor non-congruent responses identified—mainly related to item 9r (social activities and roles) and the timeframe in items 8r (fatigue) and 7r (pain intensity). Participants approached the PROMIS-10 GH through a biopsychosocial understanding of HRQOL, with one to three themes emerging per item. While certain adjustments could further improve the measure’s relevance, the limited sample size and specific pain center context restrict the generalizability of the findings. Therefore, further studies using similar methods are recommended to strengthen validation and provide more robust evidence before any revisions are made.

## Data Availability

The datasets generated and analyzed during the current study are not publicly available due to privacy regulations.

## Code availability

Not applicable. No custom code or software was used in this study.

## Declarations

### Compensation

Participants were not compensated for their participation.

### Data protection

The study followed the General Data Protection Regulation (GDPR) regarding the legal and secure handling of personal data. All data were pseudonymized, securely stored, and access was restricted to the research team.
